# Metal (II) Complexes Derived from Naphthofuran-2-carbohydrazide and Diacetylmonoxime Schiff Base: Synthesis, Spectroscopic, Electrochemical, and Biological Investigation

**DOI:** 10.1155/2014/942162

**Published:** 2014-01-30

**Authors:** R. B. Sumathi, M. B. Halli

**Affiliations:** Department of Chemistry, Gulbarga University, Gulbarga, Karnataka 585106, India

## Abstract

A new Schiff base and a new series of Co(II), Ni(II), Cu(II), Cd(II), and Hg(II) complexes were synthesized by the condensation of naphthofuran-2-carbohydrazide and diacetylmonoxime. Metal complexes of the Schiff base were prepared from their chloride salts of Co(II), Ni(II), Cu(II), Cd(II), and Hg(II) in ethanol. The ligand along with its metal complexes have been characterized on the basis of analytical data, IR, electronic, mass, ^1^HNMR, ESR spectral data, thermal studies, magnetic susceptibility, and molar conductance measurements. The nonelectrolytic behaviour of the complexes was assessed from the measured low conductance data. The elemental analysis of the complexes confirm the stoichiometry of the type CuL_2_Cl_2_ and MLCl_2_ where M = Ni(II), Co(II), Cd(II), and Hg(II) and L = Schiff base. The redox property of the Cu(II) complex was investigated by electrochemical method using cyclic voltammetry. In the light of these results, Co(II), Ni(II), and Cu(II) complexes are assigned octahedral geometry, Cd(II), and Hg(II) complexes tetrahedral geometry. In order to evaluate the effect of metal ions upon chelation, both the ligand and its metal complexes were screened for their antibacterial and antifungal activities by minimum inhibitory concentration (MIC) method. The DNA cleaving capacity of all the complexes was analysed by agarose gel electrophoresis method.

## 1. Introduction

Oxime Schiff base ligand and their metal complexes are cutting-edge areas of research due to their wide variety of applications in bioinorganic chemistry. The chemistry involving oxime moiety is quite diverse [1–3]. Oxime based ligands and their metal complexes in coordination chemistry have been increasingly expanding for their highly valued physicochemical properties, plant growth regulatory activities, reactivity patterns, and potential applications in many significant chemical processes in the field of medicine, bioorganic systems, electrochemical, and electrooptical sensors [4]. Some of the oxime complexes have also exhibited anti-carcinogenic properties [5]. It is known that oxime molecules exhibited greater potency as DNA cleavage agents [6]. Naphthofuran nuclei are key structural moieties found in a large number of biologically important natural products. Many of the natural naphthofurans, such as (±)-laevigatin [7], (+)-heritol [8, 9] and balsaminone A [10], possess interesting pharmacological and cytotoxic properties [11]. A large number of naphthofuran derivatives are endowed with various biological activities like anthelmintic, anticonvulsant, and antipyretic [12] and their plant extracts are being used for traditional medicines [13], while *mansonone D* and *Dunnione* [14] of naphthofuran family are vital biologically active agents. In addition, naphthofurans condensed with various heterocycles exhibited wide spectrum of activities [15, 16]. Coordination behaviour of metals have played an important role in the extreme fast development of inorganic chemistry and the presence of metal ions bonded to biologically active compounds may enhance their activity [17, 18]. The presence of oxygen and nitrogen donor atoms in the complexes shows significant antibacterial and anticancer activities [19].

The interaction of transition metal complexes with nucleic acids is a major area of research due to the utility of these complexes in the design and development of synthetic restriction enzymes, chemotherapeutic agents, foot printing agents, spectroscopic probes, site-specific cleavers, and molecular photo switches [20].

**Scheme 1:**
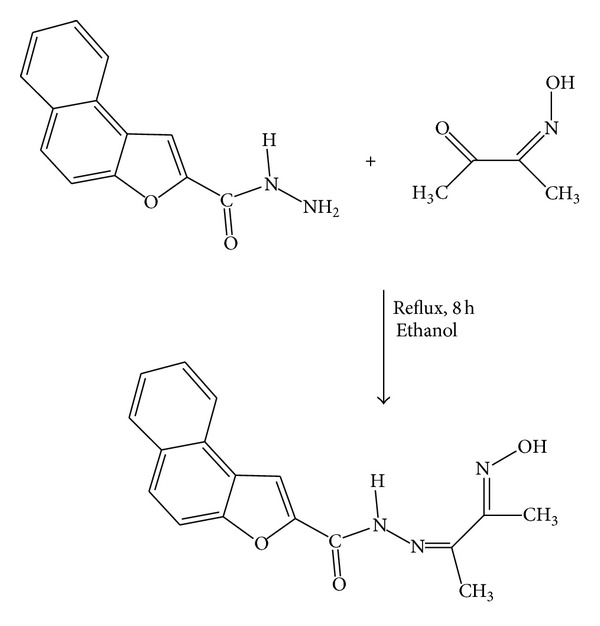
Synthesis of Schiff base ligand.

Prompted by the biological importance and applications of the above compounds in various fields, the present work is devoted to the synthesis and characterization of Schiff base ligand obtained by the condensation of naphthofuran-2-carbohydrazide with diacetylmonoxime (Scheme 1) and its metal complexes by using metal ions like Co(II), Ni(II), Cu(II), Cd(II), and Hg(II). The structural features of the Schiff base and its metal complexes were studied by various spectral analyses and screened for their antimicrobial and DNA cleavage activity studies.

## 2. Experimental

*Chemicals and Methods*. All chemicals used were of analytical reagent grade, and the solvents were distilled before use. Naphthofuran-2-carbohydrazide was synthesized according to the literature procedure [21]. The metal and chloride contents were determined as per Vogel's procedure [22]. Microanalyses (C, H, and N) were estimated out on a PerkinElmer 240C model at the Central Drug Research Institute (CDRI), Lucknow. The IR spectra of the ligand and its complexes were recorded on a PerkinElmer 783 FT-IR spectrometer in the 4000–350 cm^−1^ region in KBr pellets. The electronic spectra of the Co(II), Ni(II), and Cu(II) complexes were recorded on a ELICO SL-164 double beam UV-Visible spectrophotometer in the range of 200–1100 nm in DMF (10^−4^ M) solution. The ^1^H NMR spectra were recorded in DMSO-*d*_6_ on a BRUKER 500 MHz spectrometer using TMS as an internal reference. The ESR spectrum of the Cu(II) complex in the polycrystalline state was recorded on a Varian-E-4X band EPR spectrometer using TCNE as the “g” marker (g = 2.00277) at room temperature. The GC-MS was recorded on a JEOL GC mate mass spectrometer. The DART-MS was recorded on a JEOL-AccuTOF JMS-T100LC mass spectrometer. Thermal analyses were measured from room temperature to 1000°C in N_2_ on a Perkin Elmer, Diamond TG/DTA model thermal analyzer at STIC, Cochin, with a heating rate of 10°C min^−1^. Electrochemistry of the Cu(II) complex was recorded on a 600D series model electrochemical analyzer in DMF containing tetrabutylammonium perchlorate as the supporting electrolyte. Molar conductivity measurements were recorded on a ELICO CM-180 conductivity bridge in dry DMF (10^−3^ M) solution using a dip-type conductivity cell fitted with a platinum electrode and the magnetic susceptibility measurements were made at room temperature on a Gouy balance using Hg[Co(NCS)_4_] as the calibrant.

### 2.1. Synthesis of Schiff Base

A mixture of naphthofuran-2-carbohydrazide (0.01 mol) and diacetylmonoxime (0.01 mol) in 40 mL hot ethanolic medium was boiled under reflux for 8–10 h on a water bath. The light yellowish solid separated on partial evaporation of the solvent and subsequent cooling was filtered, washed with ethanol and finally recrystallized from ethanol. The purity of the Schiff base was checked by TLC:

 M.F = C_17_H_15_O_3_N_3_, m.p = 265°C, yield = 75%.

### 2.2. Synthesis of Co(II), Ni(II), Cu(II), Cd(II), and Hg(II) Complexes

The metal complexes were prepared using metal chlorides and the Schiff base by the general method. An ethanolic solution (40 mL) of Schiff base and Co(II), Ni(II), Cd(II), and Hg(II) chlorides in 1 : 1 molar ratio and 2 : 1 molar ratio for Cu(II) complex was refluxed on water bath for about 4 h. An aqueous solution of sodium acetate was added to the reaction mixture to adjust the pH to 6.0-7.0 and reflux was further continued for about an hour. The separated solid complexes were filtered off, washed thoroughly with water and then with little warm ethanol. The complexes obtained were finally dried under vacuum desiccator over fused CaCl_2_ (Yields: 50–55%).

## 3. Pharmacology 

### 3.1. Antibacterial and Antifungal Assays

The biological effects of all the synthesized compounds were undertaken for their antibacterial and antifungal properties by the agar and potato dextrose agar diffusion method, respectively [23, 24]. The antimicrobial activities were carried out at 100, 200, and 500 *μ*g mL^−1^ concentrations in DMSO solvent by using bacteria *E. coli* (MTCC 723), *S. aureus* (MTCC 3160), *B. subtilis* (MTCC 736), *P. aeruginosa *(MTCC 7837) and fungi, *A. flavus* (MTCC 1883), *A. niger* (MTCC 1881), *C. oxysporum* (MTCC 1777), and *C. albicans* (MTCC 3958) by minimum inhibitory concentration (MIC) method. DMSO was used as a blank and showed no activity against any of the bacterial and fungal strains.

The bacteria were subcultured on agar medium. The petri dishes were incubated at 37°C for 24 h. The fungi were subcultured on potato dextrose agar medium. The petri dishes were incubated at 37°C for 48 h. The control antibacterial (Gentamicin) and antifungal drug (Fluconazole) were also screened under similar conditions for comparison. The activity was determined by measuring the diameter of the zone showing complete inhibition.

### 3.2. DNA Cleavage Experiment

Preparation of culture media and DNA isolation of *E. coli* were done according to the literature procedure [25]. Nutrient broth (10 g L^−1^ of peptone, 5 g L^−1^ of yeast extract and 10 g L^−1^ of NaCl) was used for culturing of *E. coli*.

### 3.3. Isolation of DNA

The fresh bacterial culture (1.5 mL) is centrifuged to obtain the pellet which is then dissolved in 0.5 mL of lysis buffer (100 mM tris pH 8.0, 50 mM EDTA, 50 mM lysozyme). To this, 0.5 mL of saturated phenol was added and incubated at 55°C for 10 min, then centrifuged at 10,000 rpm for 10 min, and to the supernatant, equal volume of Chloroform: isoamyl alcohol (24 : 1), and 1/20th volume of 3 M sodium acetate (pH 4.8) was added. Then centrifuged at 10,000 rpm for 10 min and to the supernatant, 3 volumes of chilled absolute alcohol was added. The precipitated DNA was separated by centrifugation and the pellet was dried and dissolved in Tris buffer (10 mM tris pH 8.0) and stored in cold condition.

### 3.4. Agarose Gel Electrophoresis

Cleavage products were analysed by agarose gel electrophoresis method [25]. Test samples (1 mg mL^−1^) were prepared in DMF. The samples (25 *μ*g) were added to the isolated DNA of *E. coli*. The samples were incubated for 2 h at 37°C and then 20 *μ*L of DNA sample (mixed with bromophenol blue dye at 1 : 1 ratio) was loaded carefully into the electrophoresis chamber wells along with standard DNA marker containing TAE buffer (4.84 g Tris base, pH 8.0, 0.5 M EDTA/11) and finally loaded on agarose gel. A constant 50 V of electricity was then passed for around 30 min. The gel was removed and stained with 10.0 *μ*g mL^−1^ ethidium bromide solution for 10–15 min and the bands were observed under Vilber Lourmate Gel documentation system and photographed to determine the extent of DNA cleavage. The results were then compared with the standard DNA marker.

## 4. Results and Discussion

The analytical data showed that the complexes had stoichiometry of the type CuL_2_Cl_2_ and MLCl_2_ where, M = Ni(II), Co(II), Cd(II), and Hg(II) and L = Schiff base (Table 1). All the complexes were light in color, stable at room temperature, nonhygroscopic in nature, and decompose on heating. The complexes were insoluble in water and many common organic solvents but are readily soluble in DMF and DMSO. The observed molar conductance values are too low (6.85–15.80 ohm^−1^ cm^2^ mole^−1^) to account for any dissociation of the complexes in DMF, indicating their nonelectrolytic nature [26].

### 4.1. IR Spectral Studies

A comparative interpretation of the IR spectral data of the prepared Schiff base and their Co(II), Ni(II), Cu(II), Cd(II), and Hg(II) complexes are presented in Table 2.

The IR spectrum of the free Schiff base ligand exhibited a band at 3374 cm^−1^ which is assigned to *ν*(OH) of oxime group [27]. This band remains more or less at the same position in all the complexes indicating the nonparticipation of this OH group in bonding. A strong band is observed at 3239 cm^−1^ which is assigned to *ν*(NH) stretch of the CONH group. The shifting of this band to a higher wave number side in all the complexes indicated the noninvolvement of “N” of the CONH group in bonding. The strong band observed at 1686 cm^−1^ in the free ligand was attributed to *ν*(C=O) stretch of the CONH group. This band shifted to a lower wave number side in all the complexes suggesting the participation of the carbonyl oxygen in bonding with metal ions. A medium-to-strong intensity band at 1590 cm^−1^ in the free ligand was attributed to *ν*(C=N) stretch of the azomethine group [28, 29]. Coordination of the Schiff base to the metal ions through the nitrogen atom is expected to reduce electron density in the azomethine link and lower the *ν*(C=N) stretching absorption frequency. This band shifted to a lower wave number side in all the complexes indicates the participation of the azomethine nitrogen in coordination with metal ions. The medium intensity band at 1569 cm^−1^ in the free ligand was attributed to *ν*(C=N)_OX_ stretch of the oxime group [28]. This band remains almost unaltered in all the complexes suggesting the noninvolvement of nitrogen of oxime group in bonding. This indicates that carbonyl oxygen and azomethine nitrogen atoms are involved in coordination.

The assignments of bands in the far-infrared region have been controversial over the years, yet studies are useful as direct information about the metal-ligand coordination bond is obtained. The new weak intensity nonligand bands observed in the region 551–578 cm^−1^ and 428–460 cm^−1^ in the spectra of the complexes are assigned to stretching frequencies of *ν*(M–O) and *ν*(M–N) bonds, respectively. For polymeric complexes in which both terminal and bridging metal-halogen linkages are present, the **ν**(M–Cl) stretch for the terminal halide is observed at a higher wave number side than that for the bridging halide. In the present study, we assigned the weak intensity nonligand bands to the **ν**(M–Cl) stretch for the terminal halide at 333 and 356 cm^−1^ and the **ν**(M–Cl) stretch for bridging at 300 and 304 cm^−1^ for Ni(II) and Co(II) complexes, respectively, in support of their chloride-bridged polymeric structures [30, 31]. Medium intensity bands observed in the region 348–357 cm^−1^ were assigned to the **ν**(M–Cl) stretch in the Cu(II), Cd(II), and Hg(II) complexes.

### 4.2. ^1^H NMR Spectral Studies

The ^1^H NMR spectra of the ligand and its Cd(II) and Hg(II) complexes (Table 2) were recorded in DMSO-*d*_6_ and are consistent with the proposed geometry. The signal at *δ*(11.70) (s, 1H) is assigned to oxime proton of (–N–OH–) in the free ligand. This signal remains unaltered in the spectra of both the complexes confirming the noncoordination of H of (–N–OH–) in bonding with metal ions. A signal at *δ*(10.25) (s, 1H) is ascribed to the NH proton in the free ligand and shifts downfield in the region *δ*(10.62) (s, 1H) and *δ*(10.89) (s, 1H) in the Cd(II) and Hg(II) complexes, respectively, supporting the coordination of oxygen of CONH group with the metal ions. The aromatic protons at *δ*(7.60–8.50) (m, 7H) shifted downfield in the complexes. The signals at *δ*(2.10) (s, 3H) and *δ*(2.22) (s, 3H) are assigned to two methyl groups in both ligand and the complex.

### 4.3. Mass Spectral Studies

The GC-MS spectrum of the Schiff base showed a molecular ion peak M^+^ at *m*/*z* 309, equivalent to its molecular weight. The DART mass spectra of Co(II), Ni(II), and Cd(II) complexes showed a molecular ion peak M^+^ at *m*/*z* 439.16, 438.96 and 492.08, respectively, equivalent to their molecular weights. The Cu(II) and Hg(II) complexes showed a molecular ion peaks at *m*/*z* 753.15 and 581.28 which are one mass unit more than the molecular weight of the complexes, supporting the suggested structure for the complexes.

### 4.4. Electronic Spectral Studies

The electronic absorption spectra of the Co(II), Ni(II), and Cu(II) complexes were recorded in freshly prepared DMF solution (10^−4 ^M) at room temperature. The spectral data and ligand field parameters are presented in Tables 2 and 3, respectively.

The electronic spectrum of the Co(II) complex displayed bands at 14749 and 16447 cm^−1^. These two bands are assignable to ^4^T_1g_(F) → ^4^A_2g_(F) (*ν*_2_) and ^4^T_1g_(F) → ^4^T_2g_(P) (*ν*_3_) transitions, respectively, in an octahedral environment [32]. The lowest band, *ν*_1_, could not be observed due to the limited range of the instrument used but could be calculated using the band fitting procedure suggested by Underhill and Billing [33].

The Ni(II) complex exhibited two absorption bands, at 15151 and 24110 cm^−1^ assignable to ^3^A_2g_(F) → ^3^T_1g_(F) (*ν*_2_) and ^3^A_2g_(F) → ^3^T_1g_(P) (*ν*_3_) transitions, respectively, in an octahedral environment. The band *ν*_1_ was calculated by using a band fitting procedure [33].

The light green colored Cu(II) complex exhibited a single broad asymmetric band in the region 14080–16130 cm^−1^. The broadness of the band indicates the three transitions ^2^B_1g_ → ^2^A_1g_ (*ν*_1_), ^2^B_1g_ → ^2^B_2g_ (*ν*_2_), and ^2^B_1g_ → ^2^E_g_ (*ν*_3_), which are of similar energy and gives rise to only one broad absorption band. The broadness of the band may be due to dynamic Jahn-Teller distortion. All of these data suggested a distorted octahedral geometry around the Cu(II) ion. The octahedral geometry was further supported by the values of ligand field parameters, such as the Racah interelectronic repulsion parameter (B′), ligand field splitting energy (10 Dq), covalency factor (**β**), and ligand field stabilization energy (LFSE) [34].

The B′ values for the complexes were lower than the free ion values, which is an indication of the orbital overlap and delocalisation of d-orbitals. The **β** value for the Ni (II) complex was less than that of the Co(II) complex, indicating the greater covalency of the M–L bond [35].

### 4.5. Thermal Decomposition Studies

**Figure 1:**
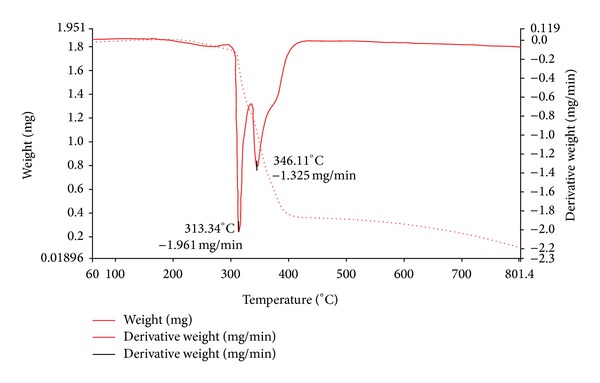
TG-DTA plot of Ni(II) complex.

Thermal stabilities were investigated for some of the complexes. The solid complexes were stable at room temperature and decompose gradually with the formation of respective metal oxides above 500°C. The nature of proposed chemical change with temperature and the percent of metal oxide obtained are given in Table 4. The spectrum of representative [Ni(C_17_H_15_O_3_N_3_)Cl_2_]_*n*_ complex presented in Figure 1 decomposes into two steps. The first step shows an endothermic peak in the temperature range of 312–314°C, which indicates the loss of two chloride molecules (Found: 15.59%, Calc: 15.95%). The weight loss in the range 345–347°C corresponds to the decomposition of the ligand (Found: 70.09%, Calc: 70.44%), further leaving behind the metal oxide residue.

In the [Cu(C_17_H_15_O_3_N_3_)_2_Cl_2_] complex, the spectrum shows three decomposition peaks, the first decomposition peak in the temperature range 209–211°C corresponds to the loss of two chloride molecules, the second decomposition peak in the temperature range 259–261°C corresponds to loss of oxime moiety, and the third decomposition peak at 438–440°C range corresponds to loss of furan moiety.

In the case of [Cd(C_17_H_15_O_3_N_3_)Cl_2_] complex, the weight losses in the temperature range 278–280°C and 389–391°C corresponds to loss of chloride and ligand molecules. The metal content in all the complexes as done by elemental analysis agrees well with the thermal studies.

### 4.6. ESR Spectrum of the Cu(II) Complex

This study gives information about the hyperfine and superhyperfine structures to elucidate the geometry and the degree of covalency of the metal-ligand bonds. The ESR spectrum of the Cu(II) complex in a polycrystalline state was recorded at room temperature show g_||_[g_||_(1) + g_||_(2) = 2.212 + 2.122] and g_⊥_ values of 2.167 and 2.038, respectively. The g_av_ was calculated to be 2.08. The spectrum showed asymmetric bands with g_||_ > g_⊥_ > 2.00277, indicating that the unpaired electrons lay predominantly in the d_x^2^−y^2^_ orbital with possible mixing of d_z^2^_ because of low symmetry [36]. The axial symmetry parameter “G” was determined as G = (g_||_ − 2.00277)/(g_⊥_ − 2.00277) = 4.66, which is more than 4 suggesting the absence of exchange coupling between Cu(II) centers in the solid state [37]. Thus, the results suggest that Cu(II) complex possesses distorted octahedral geometry [38].

### 4.7. Magnetic Studies

The magnetic moments of Co(II), Ni(II) and Cu(II) complexes were obtained at room temperature. The magnetic moment values are listed in Table 1. Under this study, the Co(II) complex exhibited the magnetic moment value of 4.82 BM indicating an octahedral geometry [39]. The magnetic moment observed for Ni(II) complex lies in the range of 2.8–3.5 BM showing a value of 2.85 which is consistent with the octahedral stereochemistry of the complex [40]. The Cu(II) complex exhibited a magnetic moment value of 1.75 BM, slightly higher than the spin-only value of 1.73 BM expected for one unpaired electron suggesting the possibility of an octahedral geometry [41].

### 4.8. Redox Study

**Figure 2:**
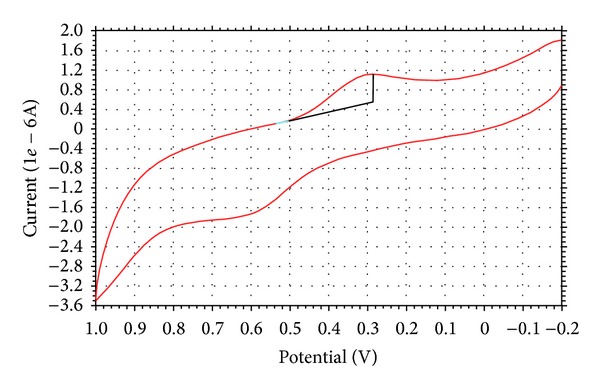
Cyclic voltammogram of Cu(II) complex.

Electron transfer plays a vital role in governing the pathway of chemical reactions. Cyclic voltammetry is the most versatile electroanalytical technique for the study of electroactive species. The cyclic voltammogram of the copper complex (Figure 2) in DMF at scan rate of 0.1 V s^−1^ using a glassy carbon working electrode shows a well-defined redox process corresponding to the formation of Cu(II)/Cu(I) couple at E_pa_ = 0.60 V versus Ag/AgCl and E_pc_ = 0.28 V. This couple is found to be quasi-reversible with ΔEp = 0.32 V and the ratio of anodic to cathodic peak currents corresponding to a simple one-electron process. The values of E_pa_ and E_pc_ of the Cu(II)/Cu(I) couple were little affected in the scan rate variation studies, suggesting no change in the quasi reversibility. The peak current increases with increasing square root of the scan rate, establishing diffusion controlled electrode process [42]. From the value of peak separation, ΔEp, and the peak current ratio we can suggest that the electrode processes are consistent with the quasi reversibility [43] of the Cu(II)/Cu(I) couple [44].

## 5. Pharmacology Results

### 5.1. Antibacterial and Antifungal Analyses

The Schiff base and its complexes used in the present study were screened against *Escherichia coli*, *Staphylococcus aureus*, *Bacillus subtilis*, *Pseudomonas aeruginosa* bacteria and *Aspergillus niger*, *Aspergillus flavus*, *Cladosporium oxysporum*, and *Candida albicans* fungi.

The microbial screening results presented in Table 5 reveal that the Schiff base is active and its metal complexes showed increased antibacterial and antifungal activity. The Co(II), Ni(II), and Cu(II) complexes exhibited good antibacterial activity against *Escherichia coli* and *Staphylococcus aureus* whereas *Bacillus subtilis* and *Pseudomonas aeruginosa* have shown moderate activity. In the case of antifungal activity all the tested complexes have shown good activity against both *Aspergillus niger* and *Aspergillus flavus* fungi and moderate activity against* Cladosporium oxysporum* and *Candida albicans*. The Cd(II) and Hg(II) complexes have shown higher activity against both the bacterial and fungal strains compared to the Schiff base ligand and Co(II), Ni(II), and Cu(II) complexes, however, are less active than the standard drugs. Generally, it is claimed that the enhancement in the activity may be due to the fact that the ligand possess C=N bond. The ligands with nitrogen and oxygen donor atoms inhibit enzyme activity, since the enzymes which require these groups for their activity appear to be especially more susceptible to deactivation by metal ions on coordination. The activity of the metal chelates can be explained on the basis of Overtone's concept and Tweedy's chelation theory [45]. The enhanced activity of the metal complexes may be retained to the increased lipophilic nature of the complexes which arose from the chelation. It was also noted that the toxicity of the metal complexes increases on increasing the metal ion concentration. In general, metal complexes are more active than the ligands because metal complexes may serve as a vehicle for activation of ligands as the principle cytotoxic species [46]. However, from the data, it is inferred that the newly synthesized compounds are effective fungicides than bactericides.

### 5.2. Electrophoretic Analysis

**Figure 3:**
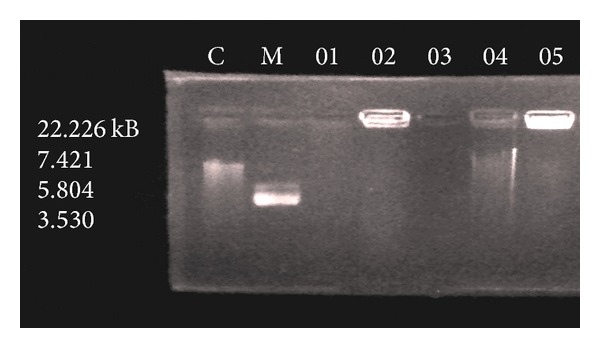
DNA cleavage activity studies of Cu(II), Ni(II), Co(II), Cd(II), and Hg(II) (lanes-O1, O2, O3, O4, and O5) complexes, respectively. M: standard molecular weight Marker; C: control DNA.

The Co(II), Ni(II), Cu(II), Cd(II), and Hg(II) complexes were monitored for their DNA cleavage ability by agarose gel electrophoresis method against DNA of *E. coli* at 100 *μ*g mL^−1^ (Figure 3).

The cleavage efficiency of the complexes compared to that of the control is due to their efficient DNA-binding ability. DNA-binding studies helps in the rational designing and construction of new and more efficient drugs targeted to DNA [47]. A comparative study of gel electrophoresis analysis of the control DNA with the metal complexes revealed that the control DNA does not show any cleavage whereas Cu(II) and Co(II) complexes (lanes O1 and O3) have shown complete cleavage of DNA, while the Ni(II), Cd(II), and Hg(II) complexes (lanes O2, O4, and O5) have exhibited partial cleavage activity, which is evidenced by diminishing in intensity of the lanes. However, the nature of reactive intermediates involved in the DNA cleavage by the complexes is not clear. The results indicated the important role of metal ions in isolated DNA cleavage reactions. As the complexes were observed to cleave the DNA, it can be concluded that, the compounds inhibit the growth of the pathogenic organism by cleaving the genome [48]. The different DNA cleavage ability of the complexes may be due to the different binding affinity of the complexes to the DNA molecule.

## 6. Conclusions

**Figure 4:**
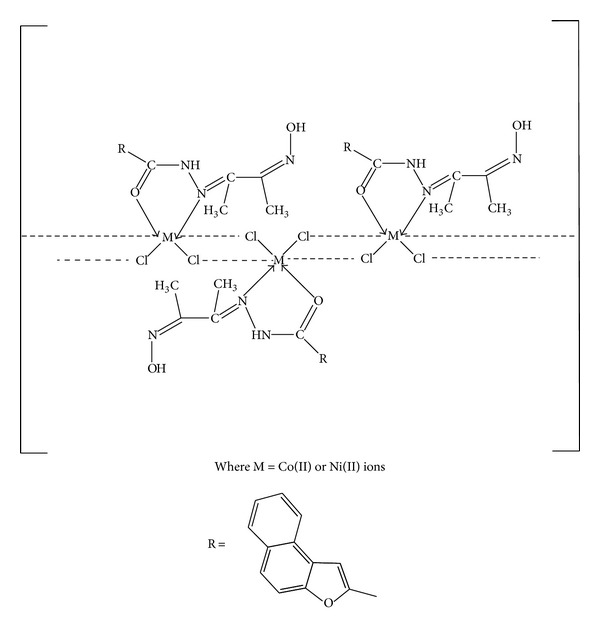
Suggested structure for [Co(C_17_H_15_O_3_N_3_)Cl_2_]_*n*_ or [Ni(C_17_H_15_O_3_N_3_)Cl_2_]_*n*_ complexes.

**Figure 5:**
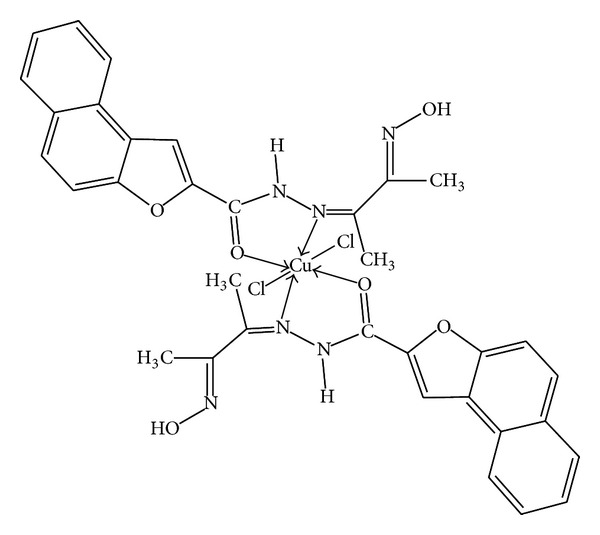
Suggested structure for [Cu(C_17_H_15_O_3_N_3_)_2_Cl_2_] complex.

**Figure 6:**
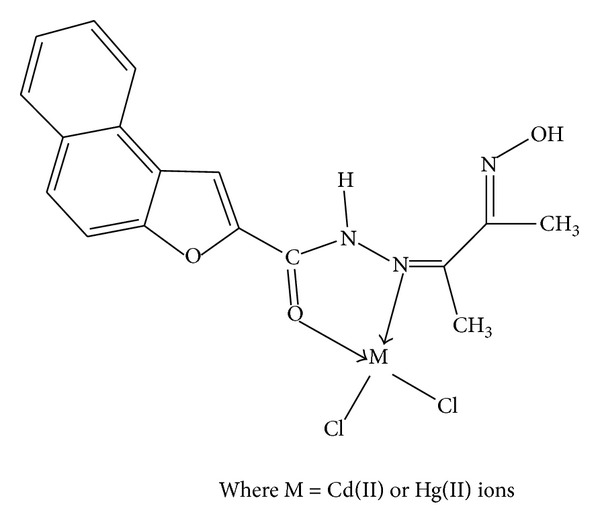
Suggested structure for [Cd(C_17_H_15_O_3_N_3_)Cl_2_] or [Hg(C_17_H_15_O_3_N_3_)Cl_2_] complexes.

In this research article, we have synthesized a new Schiff base ligand and its metal complexes. The formation of the compounds has been confirmed by the analytical data, IR, electronic, mass, ^1^H NMR, thermal and ESR spectral studies, magnetic susceptibility, electrochemical, and molar conductance data. The above studies reveal that the Schiff base acts as neutral bidentate coordinating through azomethine nitrogen and carbonyl oxygen atoms to the metal ions. The electrochemical behavior of Cu(II) complex exhibited the one electron transfer quasi-reversible redox couple. The antimicrobial activity results displayed that Cd(II) and Hg(II) complexes exhibit higher activity compared to Co(II), Ni(II), and Cu(II) complexes. It is well noticed that the synthesized metal complexes exhibited more inhibitory effects than the parent Schiff base ligand as the efficacy of the organic compound is positively modified upon coordination with the metal ions. The gel electrophoresis experiment depicted that Cu(II) and Co(II) complexes cleaved the DNA molecule completely. Thus, the present study gives valuable information to the bioorganic/inorganic chemists and contributes better knowledge to the field of bioinorganic chemistry. On the basis of their physicochemical data, we propose polymeric octahedral geometry for Co(II) and Ni(II) complexes (Figure 4), dimeric octahedral for Cu(II) complex (Figure 5), and tetrahedral structures to Cd(II) and Hg(II) complexes (Figure 6).

## Figures and Tables

**Table 1:** Physical and analytical data of Schiff base and its complexes.

Compounds	Mol. Wt	m.p (°C)	C%	H%	N%	M%	Cl%	Λ_M_*	*μ*_eff_ (BM)
			Found (Calcd)	Found (Calcd)	Found (Calcd)	Found (Calcd)	Found (Calcd)		
C_17_H_15_O_3_N_3_	309.11	265	65.72 (65.97)	4.66 (4.83)	13.31 (13.56)	—	—	—	—
[Co(C_17_H_15_O_3_N_3_)Cl_2_]_*n*_	439.04	>300	46.22 (46.45)	3.23 (3.40)	9.21 (9.54)	13.35 (13.41)	16.03 (16.15)	8.10	4.82
[Ni(C_17_H_15_O_3_N_3_)Cl_2_]_*n*_	438.80	>300	46.21 (46.47)	3.19 (3.39)	9.32 (9.55)	13.28 (13.36)	16.03 (16.18)	6.85	2.85
[Cu(C_17_H_15_O_3_N_3_)_2_Cl_2_]	752.72	285	53.92 (54.17)	3.75 (3.96)	10.82 (11.13)	8.32 (8.41)	9.26 (9.41)	12.40	1.75
[Cd(C_17_H_15_O_3_N_3_)Cl_2_]	492.52	290	41.21 (41.40)	2.99 (3.02)	8.32 (8.51)	22.75 (22.81)	14.31 (14.40)	15.80	—
[Hg(C_17_H_15_O_3_N_3_)Cl_2_]	580.70	298	35.01 (35.12)	2.34 (2.57)	7.05 (7.21)	34.42 (34.53)	12.10 (12.21)	11.40	—

*Molar conductance values in ohm^−1^cm^2^mole^−1^.

**Table 2:** Spectral data of all the newly synthesized compounds.

Compounds	Spectral data
C_17_H_15_O_3_N_3_	IR, *ν*/cm^−1^ (KBr): 1569 (C=N)_OX_, 1590 (C=N), 1686 (C=O), 3239 (NH), 3374 (OH)_OX_ ^1^H NMR, (*δ*) (*d*_6_-DMSO): 2.10 (s, 3H, CH_3_), 2.22 (s, 3H, CH_3_), 7.60–8.50 (m, 7H, Ar), 10.25 (NH), 11.70 (–N–OH) GC-MS, *m*/*z* (*I*_*r*_/%): 309 (100%)

[Co(C_17_H_15_O_3_N_3_)Cl_2_]_*n*_	IR, *ν*/cm^−1^ (KBr): 445 (M–N), 561 (M–O), 1568 (C=N)_OX_, 1566 (C=N), 1662 (C=O), 3246 (NH), 3374 (–N–OH) DART-MS, *m*/*z* (*I*_*r*_/%): 439.16 (40%) UV-Vis (DMF, cm^−1^): 6902, 14749, 16447

[Ni(C_17_H_15_O_3_N_3_)Cl_2_]_*n*_	IR, *ν*/cm^−1^ (KBr): 460 (M–N), 551 (M–O), 1569 (C=N)_OX_, 1561 (C=N), 1651 (C=O), 3249 (NH), 3375 (–N–OH) DART-MS, *m*/*z* (*I*_*r*_/%): 438.96 (30%) UV-Vis (DMF, cm^−1^): 7590, 15151, 24110

[Cu(C_17_H_15_O_3_N_3_)_2_Cl_2_]	IR, *ν*/cm^−1^ (KBr): 450 (M–N), 553 (M–O), 1569 (C=N)_OX_, 1552 (C=N), 1656 (C=O), 3244 (NH), 3374 (–N–OH) DART-MS, *m*/*z* + 1 (*I*_*r*_/%): 753.15 (25%) UV-Vis (DMF, cm^−1^): 14080–16130

[Cd(C_17_H_15_O_3_N_3_)Cl_2_]	IR, *ν*/cm^−1^ (KBr): 458 (M–N), 563 (M–O), 1568 (C=N)_OX_, 1565 (C=N), 1644 (C=O), 3252 (NH), 3375 (–N–OH) ^1^H NMR, *δ* (*d*_6_-DMSO): 2.10 (s, 3H, CH_3_), 2.22 (s, 3H, CH_3_), 7.62–8.53 (m, 7H, Ar), 10.62 (NH), 11.70 (–N–OH) DART-MS, *m*/*z* (*I*_*r*_/%): 492.08 (40%)

[Hg(C_17_H_15_O_3_N_3_)Cl_2_]	IR, *ν*/cm^−1^ (KBr): 428 (M–N), 578 (M–O), 1568 (C=N)_OX_, 1571 (C=N), 1647 (C=O), 3251 (NH), 3374 (–N–OH) ^1^H NMR, *δ* (*d*_6_-DMSO): 2.10 (s, 3H, CH_3_), 2.22 (s, 3H, CH_3_), 7.63–8.53 (m, 7H, Ar), 10.89 (NH), 11.70 (–N–OH) DART-MS, *m*/*z* + 1 (*I*_*r*_/%): 581.28 (40%)

**Table 3:** Electronic spectral bands and ligand field parameters of the Co(II), Ni(II), and Cu(II) complexes in DMF (10^−4^ M) solution.

Complexes	Transitions in cm^−1^			Dq (cm^−1^)	B′ (cm^−1^)	*β*	*β*%	*ν*_2_/*ν*_1_	LFSE (kcal)
	*ν*_1_*	*ν*_2_	*ν*_3_						
[Co(C_17_H_15_O_3_N_3_)Cl_2_]_*n*_	6902	14749	16447	784	699	0.720	28.01	2.136	13.44
[Ni(C_17_H_15_O_3_N_3_)Cl_2_]_*n*_	7590	15151	24110	963	689	0.662	33.75	1.573	33.01
[Cu(C_17_H_15_O_3_N_3_)_2_Cl_2_]	14080–16130			1379	—	—	—	—	23.64

*Calculated values.

**Table 4:** Thermogravimetric data of Ni(II), Cu(II), and Cd(II) complexes.

Empirical formulae of the complexes	Decomposition temperature (°C)	Weight loss (%)		Metal oxide (%)		Inference
		Found	Calc.	Found	Calc.	
[Ni(C_17_H_15_O_3_N_3_)Cl_2_]_*n*_	312–314	15.59	15.95	13.12	13.61	Loss of chloride molecules
	345–347	70.09	70.44			Loss of ligand

[Cu(C_17_H_15_O_3_N_3_)_2_Cl_2_]	209–211	8.95	9.29	8.14	8.43	Loss of chloride molecules
	259–261	22.23	22.61			Loss of oxime moiety
	438–440	59.10	59.51			Loss of furan moiety

[Cd(C_17_H_15_O_3_N_3_)Cl_2_]	278–280	13.85	14.21	22.85	23.02	Loss of chloride molecules
	389–391	62.32	62.76			Loss of ligand

**Table 5:** Antimicrobial activity results of the Schiff base and its metal complexes (MIC).

Schiff base/complexes	Conc. (*μ*g mL^−1^)	Zone of inhibition against bacteria and fungi (mm)							
		*E. coli *	*S. aureus *	*B. subtilis *	*P. aeruginosa *	*A. niger *	*A. flavus *	*C. oxysporum *	*C. albicans *
C_17_H_15_O_3_N_3_	100	05	05	—	04	07	07	06	—
	200	12	13	09	10	13	14	12	08
	500	14	15	11	13	15	16	13	14

[Co(C_17_H_15_O_3_N_3_)Cl_2_]_*n*_	100	06	07	04	05	09	08	07	06
	200	14	14	10	12	14	16	13	09
	500	16	16	12	14	16	18	15	15

[Ni(C_17_H_15_O_3_N_3_)Cl_2_]_*n*_	100	07	08	04	06	09	10	08	07
	200	14	15	10	13	15	16	14	16
	500	15	16	12	13	16	19	15	15

[Cu(C_17_H_15_O_3_N_3_)_2_Cl_2_]	100	07	07	05	06	10	11	09	06
	200	15	17	13	15	16	17	15	17
	500	16	18	14	17	17	19	15	16

[Cd(C_17_H_15_O_3_N_3_)Cl_2_]	100	11	13	08	10	18	19	17	16
	200	19	18	15	16	20	20	19	18
	500	22	21	18	19	21	22	20	22

[Hg(C_17_H_15_O_3_N_3_)Cl_2_]	100	11	12	09	11	18	20	17	19
	200	20	20	16	17	21	22	18	19
	500	23	21	17	20	25	26	24	22

Gentamicin	500	29	28	25	26	—	—	—	—

Fluconazole	500	—	—	—		30	31	30	29

Note: less than 12 mm: inactive; 12–16 mm: moderately active; above 18 mm: highly active.
